# Cheese whey to biohydrogen and useful organic acids: A non-pathogenic microbial treatment by *L*. *acidophilus*

**DOI:** 10.1038/s41598-019-42752-3

**Published:** 2019-06-06

**Authors:** Anjana Pandey, Saumya Srivastava, Priya Rai, Mikel Duke

**Affiliations:** 1 Department of Biotechnology, Motilal Nehru National Institute of Technology (MNNIT) Allahabad, Prayagraj, (UP) India; 2 Institute for Sustainable Industries and Liveable Cities, Victoria University, Melbourne, Australia

**Keywords:** Biochemistry, Microbiology

## Abstract

The burgeoning organic waste and continuously increasing energy demands have resulted in significant environmental pollution concerns. To address this issue, the potential of different bacteria to produce biogas/biohydrogen from organic waste can be utilized as a source of renewable energy, however these pathogenic bacteria are not safe to use without strict contact isolation. In this study the role of safe food grade lactic acid bacteria (*Lactobacillus spp*.) was investigated for production of biogas from cheese waste with starting hexose concentration 32 g/L. The bacterium *Lactobacillus acidophilus* was identified as one of the major biogas producers at optimum pH of 6.5. Further the optimum inoculum conditions were found to be 12.5% at inoculum age of 18 h. During the investigation the maximum biogas production was observed to be 1665 mL after 72 hours of incubation at pH 6.5. The biogas production was accompanied with production of other valuable metabolites in the form of organic acids including pyruvate, propionate, acetate, lactate, formate and butyrate. Thus this research is paving way for nonpathogenic production of biohydrogen from food waste.

## Introduction

Exhaustion of conventional energy reserves, global warming and elevated environmental pollution have necessitated the utilization of alternative energy sources^1,2^. The utmost challenge today is the substitution of conventional fuels with renewable and carbon neutral energy sources. One such possibility to overcome this challenge is the use of microorganisms that are capable to produce energy from renewable sources, and in sufficient quantity to substitute energy produced by fossil fuels, without disturbing the environmental equilibrium and human food-supply system^3–8^.

As per FAO report^9^, approximately 1.3 billion tonnes of foods including fresh vegetables, fruits, meat, bakery and dairy products are wasted in the food supply chain. The quantity of wasted food discharged from different places such as food processing plants, cafeterias, restaurants and household kitchens is expected to upsurge in the coming decades owing to population and economic growth. The wasted food can be utilised, along with other combustible municipal wastes, for energy production. Despite this relatively simple means to harness energy from waste, combustion of food waste results in air pollution and loss of chemical values of organic materials. Studies have suggested that a proper food management of food waste is needed^10^.

Food waste with high content of carbohydrate is suitable for H_2_ production which is a carbon free energy carrier that can be used for clean energy production. The hydrogen (H_2_) yields ranging from 0.9 to 8.35 mol H_2_/mol hexose^11^ have been observed. Numerous factors including food waste composition, pre-treatment methods and process used can affect H_2_ production. Hydrogen production from carbohydrate based waste has been observed to be 20 fold higher than hydrogen yield from fat and protein based food waste^12^.

Hydrogen is regarded as a substitute energy carrier owing to its greater energy yield (122 kJ/g) i.e. 2.75 times higher than that of common hydrocarbon based fuels and non-polluting emissions. H_2_ can be produced by dark fermentation using organic wastes as starting material^13^.

Hydrogen gas exerts an important position due to its noncarcinogenic character resulting in cleaner and sustainable energy system. It can be produced from fossil fuels or any other source which is carbon free leading to reduce the emission of greenhouse gases^14^. More than 90% of hydrogen production worldwide is being executed by means of fossil fuels. Several microorganisms have the enzyme hydrogenases which help to release molecular hydrogen by oxidizing hydrogen to electrons and protons. The common way to produce hydrogen biologically is microbial fermentation i.e. by decomposition of organic substrates to hydrogen and carbon dioxide^15^. The bacteria which are able to produce hydrogen have the capability to grow autotrophically by utilizing the hydrogen gas as an energy substrate and oxygen as a terminal electron acceptor for production of water as end product^16^. Variety of biological species are involved in production of hydrogen viz. cyanobacteria, fermentative and photosynthetic bacteria etc^17^.

The estimated yearly production of lignocellulosic biomass is nearly 2.20 × 10^12^ Kg (dry weight) from agriculture and forestry residuals, energy crops, aquatic plants and algae^18^ which have potential to produce H_2_ at very low costs by hydrolysis and fermentation^19^. Translating the energy from these wastes into valuable energy offers two concurrent benefits: the production of energy and reduction in environmental pollution. Diverse groups of anaerobic microorganisms possess the potential to break complex organic matter into three valuable energy outputs, which can be garnered proficiently: methane gas (CH_4_), hydrogen gas (H_2_) and electrons produced by a microbial fuel cell (MFC)^6–8^.

The different reactor conditions reported use of different bacteria capable of bioconversion of organic wastes into hydrogen and most of the seed cultures used include pathogenic strains of bacteria and required specific growth conditions. The application of food grade bacteria including *Lactobacillus* and *Lactococcus* can help in overcoming these problems by introducing non-pathogenic way of waste predisposal treatment and energy production. Hence, in this study first time the effects of different food grade bacteria on biohydrogen production have been analysed, along with the effects of different parameters such as inoculum conditions and pH. Based on the previous reports it can be hypothesized that food grade bacteria will produce the biohydrogen comparable to that from pathogenic bacteria. The proposed hypothesis was studied by analysis of biohydrogen production from food wastes by using non-pathogenic bacteria. Different parameters such as pH, inoculum age and size have been optimized to get the maximum biohydrogen production. Cheese whey has been used as a complex substrate for biohydrogen production by *Lactobacillus acidophilus* in a batch reactor of capacity 2 litres.

## Materials and Methods

### Seed Culture

The standard strains of *Lactobacillus acidophilus* (Non-pathogenic, ATCC 4356), *Lactobacillus casei* (Non-pathogenic, ATCC 393), *Lactobacillus paracasei* (Non-pathogenic, ATCC BAA52), *Lactococcus lactis* (Non-pathogenic, ATCC 19435) and *E*. *coli* have been used in this study. Single colony obtained from well grown plate containing M17 agar plate supplemented with 1% dextrose was used for inoculum preparation.

### Comparison of microbes for evaluation of biohydrogen production potential

All the microbes were screened for hydrogen producing capability by conducting batch experiments in triplicates under anaerobic conditions. The medium composition remains the same as mentioned above. All the experiments were conducted using polypropylene bottles (capacity 150 mL) with working volume of 100 mL. These bottles were tightly capped with butyl rubber stopper to make it air tight and the whole setup was made anaerobic by sparging of argon gas. For the collection of gas evolved during the fermentation, the set up was equipped with disposable air tight syringes. The sealed bottles were carefully placed in incubator shaker (100 rpm) maintaining temperature at 37 °C. The gas collected was measured by water displacement method.

### Optimization experiments of growth conditions for hydrogen production

1. **Initial pH:** The experiments were conducted to optimize the initial medium pH taking five different values 5.5, 6.0, 6.5, 7.0 and 7.5 knowing the fact that optimal pH for dark fermentative hydrogen production mostly falls within the range of 5–8. The experiments were conducted in triplicates to minimise the manual error for 18 h at 37 °C. The nutrient media composition containing M17 broth supplemented with 1% (w/v) glucose was used. An inoculum size was 10% v/v of total working volume.
2. **Inoculum Age:** To optimize the inoculum age, experiments were performed under the same experimental conditions with variation in inoculum age i.e. 10 h, 12 h,14 h,16 h,18 h and 20 h keeping rest other conditions same as mentioned above at 37 °C at optimum pH i.e. 6.5.
3. **Inoculum size:** After the optimization of initial medium pH and inoculums age experiments were performed to optimize the inoculum size. All the experiment conditions were same as that of the pH optimization experiments except of the variation in the inoculum size taking the values as 20, 15, 13, 12 and 10 (all in percentage v/v) at 37 °C. The pH and inoculum age were kept at optimum value i.e. 6.5 and 18 h respectively.

### Reactor study

Experiment was carried out in the Biostat A^+^ fermenter under batch mode. The volume of reactor vessel was 2 L, containing 900 mL media (3% cheese whey, 2.5 g MRS media, 2.5 g of M17 media, 2.5 g of yeast extract and anamox nutrient solution) maintaining the pH at 6.5 with 20% of 18 h grown inoculum of *L*. *acidophilus*. The conditions for this fermentation were maintained with 0% oxygen, stirring at 150 rpm and temperature at 37 °C. The gas produced during the experiments was collected by water displacement method and samples were withdrawn at different time for metabolite analysis.

### Analytical methods

The gaseous products (CO_2_ and H_2_) evolved from fermentation experiments were analysed by Drager’s tube and gas chromatograph (Agilent 7890 A) having a thermo conductivity detector and capillary column (HP-PLOT/Q). Nitrogen gas served as carrier and pure hydrogen served as standard. The oven temperature was 90 °C and temperature of detector and injector was 100 °C and 70 °C respectively.

The fermentation effluent collected at different time during experiment were analysed by using HPLC (Shimadzu) fitted with column Animex HPX-87 H (S. N0. 31262 and diameter 300 × 7.8 mm) using 0.018 M H_2_SO_4_ with a flow rate of 0.7 mL/min at 65 °C for 30 minutes.

### Statistical analysis

The one way ANOVA was performed to test the significance of the experimental data using Microsoft Excel. During the ANOVA analysis the results of test samples were compared with the control samples and analysed for significance level of 95% (*p* < *0*.*05*).

## Result and Discussion

### Evaluation of food grade bacterial strains for biogas production

**Figure 1:**
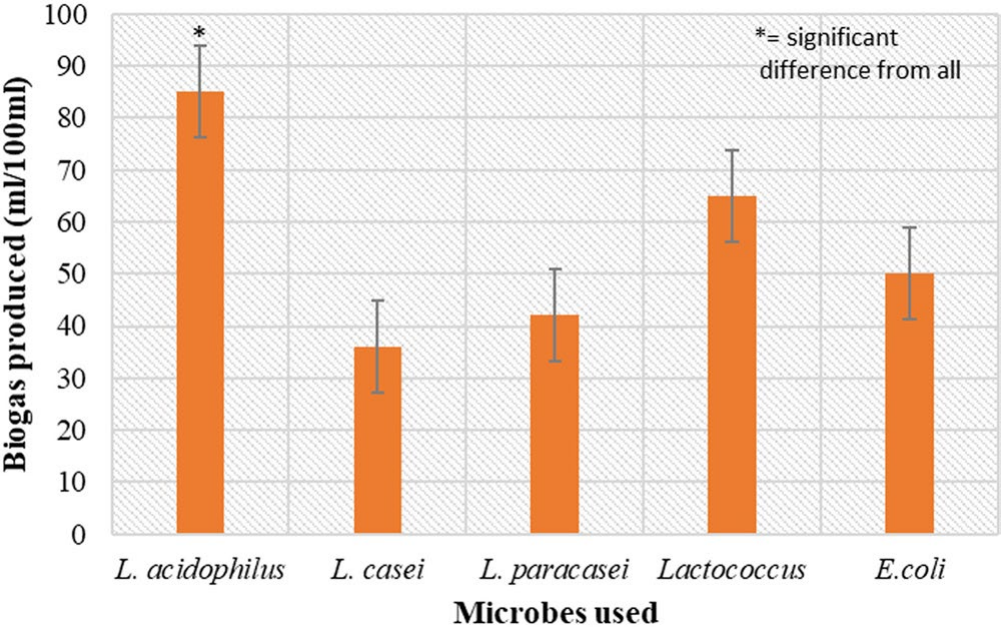
Comparison of biogas production by different bacterial strains in batch mode at 37 °C.

The selected strains were analysed for biogas production and the results are represented in Fig. (1). From the data it can be observed that *L*. *acidophilus* was most efficient in biogas production with a value of 85 mL/100 mL of culture media used, whereas *L*. *casei* was observed to be least efficient with a production value of 36 mL/100 mL. The other three strains used viz. *L*. *paracasei*, *Lactococcus lactis* and *E*. *coli* were moderate producers of biogas with production values of 42 mL/100 mL, 65 mL/100 mL and 50 mL/100 mL respectively. In a study, the waste bread hydrolysate was used as substrate for biohydrogen production by *Biohydrogenbacterium* R3. The maximum H_2_ yield of 103 mL H_2_/g waste bread was observed^20^. In another experiment, effective hydrogen production from food waste hydrolysate in different continuous mixed immobilized sludge reactors (CMISRs) at packing ratios of 10%, 15% and 20% have been reported. The biohydrogen production at a packing ratio of 15% was highest with a hydrogen production rate of 353.9 mL/h/L and at high organic loading rate of 40 kg/m^3^/d^21^. Anaerobic digestion of food waste by using *Escherichia cloacae* and *Enterobacter aerogenes* had produced biohydrogen of 155.2 mL/g of volatile solids (VS)^22^. In a continuous stirred tank reactor (CSTR) provided with sugarcane, 3.38 mmol H_2_/L/h hydrogen were produced by using *Clostridium butyricum*^23^. Configurations including membrane filtration enhanced H2 yield by over 300% compared to a more conventional stirred reactor^24^.

### Effect of pH on biogas production

**Figure 2:**
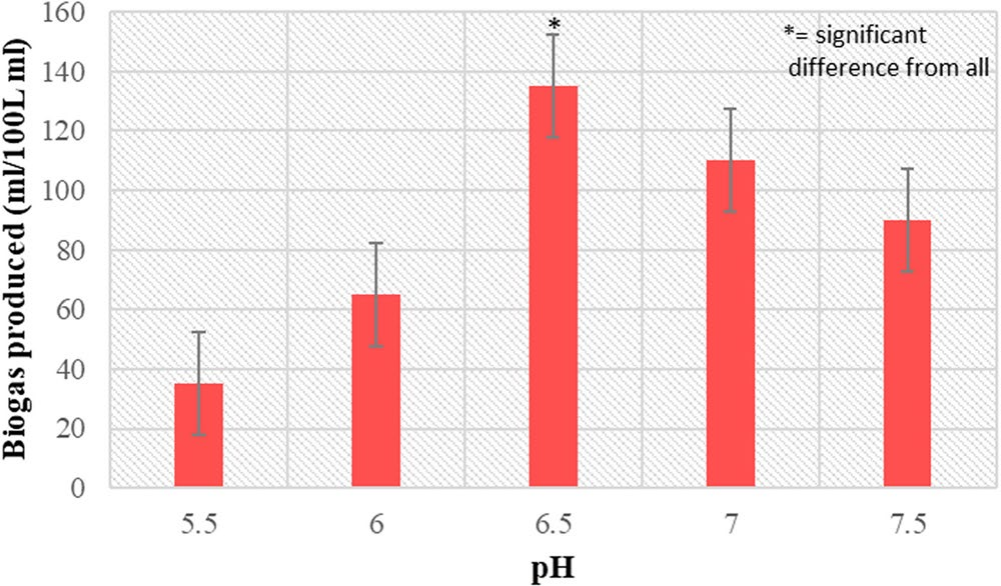
Effect of pH (*p* < 0.05) on biogas production by food grade bacteria *L*. *acidophilus* in the batch mode at 37 °C.

The effect of pH on biogas production by *L*. *acidophilus* is represented in Fig. 2. The biogas production was observed at different starting pH values of 5.5, 6.0, 6.5, 7.0 and 7.5. From the results it has been observed that the biogas production initially increased up to pH 6.5 with a production value of 135 ml/100 ml. Above this pH, biogas production started decreasing. Therefore, it can be concluded that pH 6.5 is optimal for biogas production under these experimental conditions. By using coconut milk wastewater as the substrate, maximum biogas production i.e. 0.28 L H_2_/L has been reported at pH 6.5 indicating that such parameter is crucial for dark fermentative hydrogen production^25^. Activity of enzymes varied according to different pH conditions that become a very important factor for stimulating the biohydrogen producing ability of microrganisms.

Among the studied fermentation pH values, relatively high biogas yield was observed at pH 6.5 (135 mL/100 mL). Next higher values of biogas produced was observed at pH 7.0 (110 mL/100 mL), whereas biogas production in fermentation at acidic pH values decreased more substantially. This decrease in biogas production at lower pH values can be attributed to decline in the system pH values below 5.0 due to acid production, that results in shifting of metabolic pathway to solventogenesis from acidogenesis leading to H_2_ production suppression. The data obtained in this study is in accordance with other reports, showing maximum biogas/H_2_ production at pH values of 6.0 by use of anaerobic mixed microflora^26^, pH 6.5 by use of sewage microflora^25^. The slight variation in optimum pH values in different studies can be attributed to change in seed culture composition, inoculum conditions and culture conditions.

### Effect of inoculum age and volume

**Figure 3:**
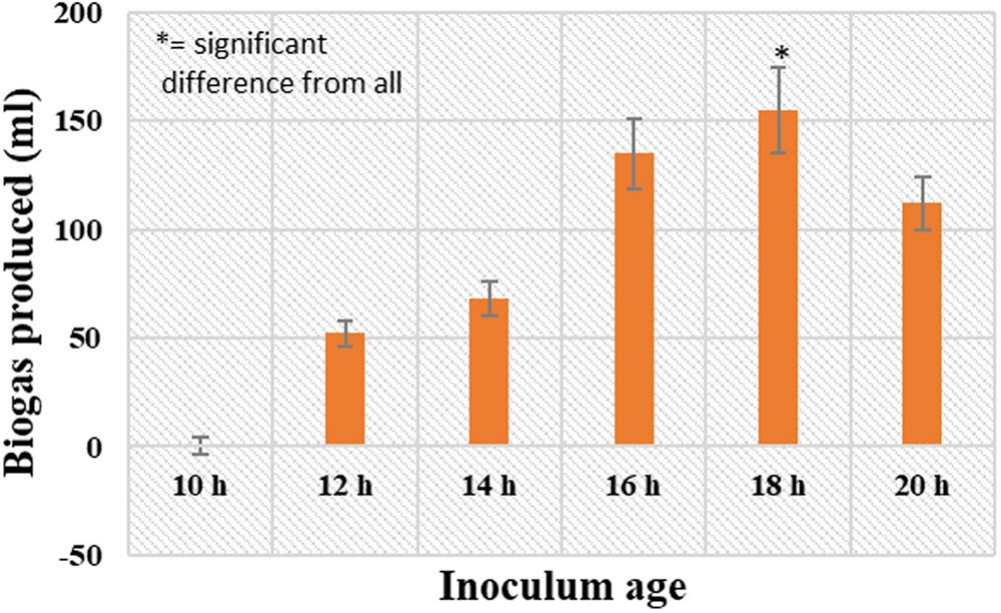
Effect of inoculum age (*p* < 0.05) on biogas production in the batch mode from *L*. *acidophilus* at pH 6.5 and temperature 37 °C.

Further the effect of inoculum conditions viz. inoculum age and inoculum volume were investigated. To study the effect of inoculum age the inoculum from cultures incubated for different time intervals were used. Figure 3 represents the effect of inoculum age on biogas production. The results show that initially the biogas production increased with increase in inoculum up to 18 h, attaining a maximum value of 155 ml. The next sample was at 20 h, where showed the biogas production decreasing again. Therefore, it can be inferred from the results that 18 h is the optimum inoculum age for biogas production.

The experiment was performed to examine the inoculum age effect on hydrogen production by using the inoculum grown for 10, 12, 14, 16, 18 and 20 h.

The results showed the strong relationship among inoculum age and healthy growth of the culture alongside H_2_ production. The inoculum with 18 h age exhibited the highest percentage of biogas (155 mL/L) production. The large retention times might be responsible for the bacterial metabolism to drive in the synthesis of other metabolites such as Poly-Beta-Hydroxybutyrate (PHB) alongside the H_2_ production^27^. The loss of H_2_ production activity in batch cultures of *R*. *sphaeroides* S was associated with the declined activity of the electron carrier ferredoxin in time dependant manner^28,29^. For PNS bacteria, an inoculum from the exponential growth phase is most suitable for elevated yield of H_2_^30^. All the studies are highlighting the effect of inoculum age on different strains with a common conclusion of declined hydrogen yield with increased time span and this study also demonstrates that H_2_ production by food grade bacteria can be achieved maximum at the inoculum age of 18 h and lowered with increase in inoculum age.

**Figure 4:**
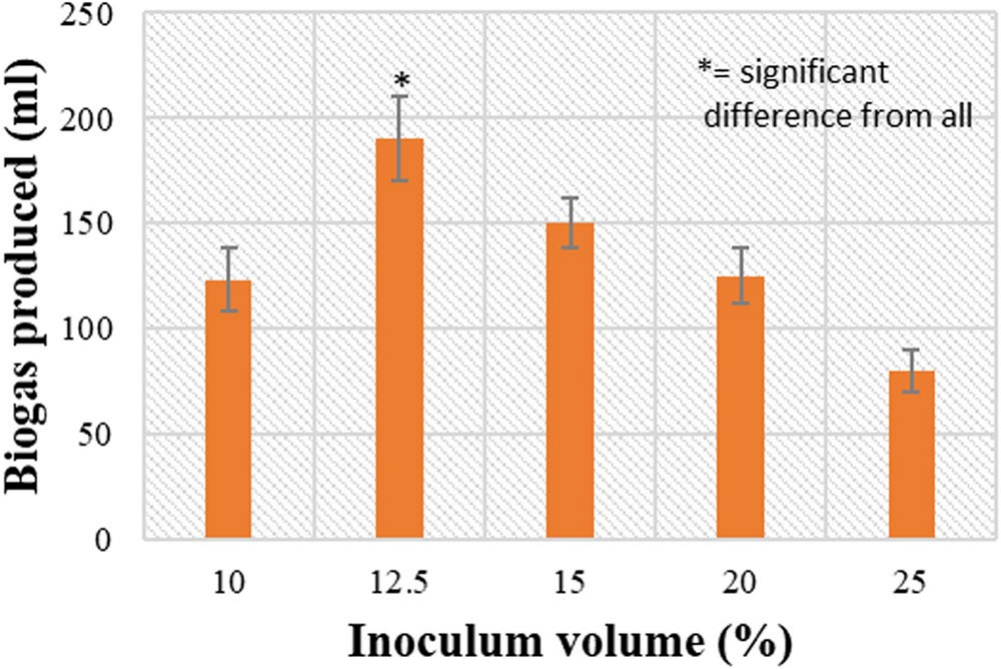
Effect of inoculum volume (*p* < 0.05) on biogas production from *L*. *acidophilus* at pH 6.5 and 37 °C in the batch mode fermentation.

After that the effect of inoculum volume on biogas production was analysed by varying the volumes of inoculum used, from the results shown in Fig. 4, it can be observed that initially the biogas production increased with increase in inoculum volume and attained a maximum value of 190 mL at 12.5% inoculum volume and then further started to decrease in concentration dependent manner with minimum biogas production of 80 mL at 25% inoculum volume thus indicating that 12.5% inoculum volume is optimum for biogas production.

The inoculum volume also plays an imperative role in biogas/H_2_ production. 10%, 12.5%, 15%, 20%, 25% were studied as different inoculum volumes. The results exhibit that the most productive inoculum volume was 12.5% to produce 190 mL of biogas but at lower and higher inoculum volumes the biogas production decreased. In a study, it has been observed that the H_2_ yield improved from 2 mmol h^−1^ at 1% inoculum size to 2.36 mmol h^−1^ with 10% inoculum size but the highest yield was achieved with 1% inoculum^31^ signifies a high substrate to cell ratio would extend the growth phase and provide a longer duration of high rates of H_2_ production. However, in another study it has been reported that a low substrate (cellulose) to cell density enabled higher H_2_ yield by using a mixed H_2_ producing culture^32^. The decline in total gas or H_2_ at the end of the metabolic process can be credited to the consumption of gas by bacteria as the collected gas was directly in contact with the culture without any trap system.

### Biogas production in batch fermentation and sugar utilization

**Figure 5:**
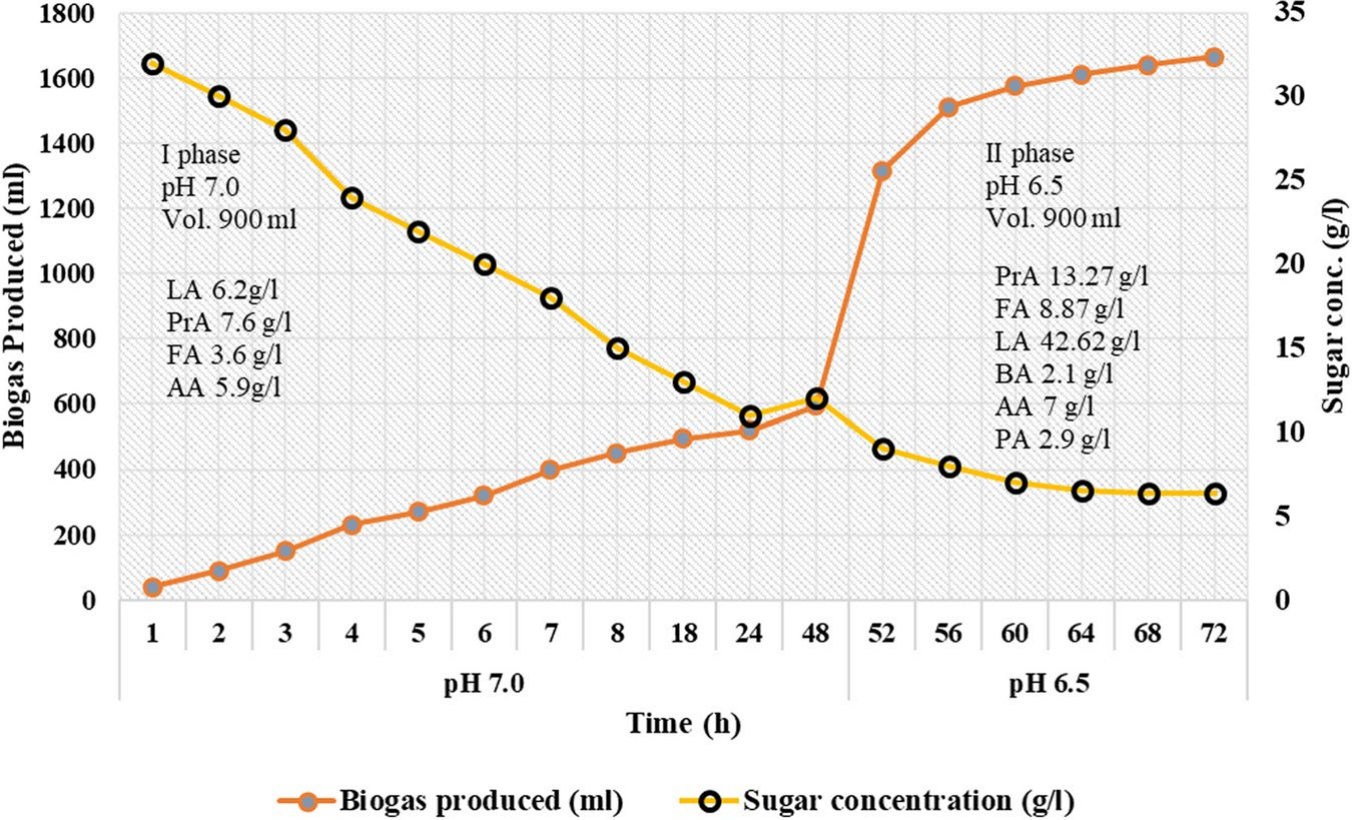
Kinetics of biogas and organic acids production with sugar utilization in 2 phases i.e. phase I (pH 7.0) and phase II (pH 6.5) from *L*. *acidophilus* at 37 °C (LA = lactic acid, BA = butyric acid, AA = acetic acid, PA = pyruvic acid, FA = formic acid, PrA = propionic acid).

The kinetics of biogas production with sugar utilization was observed during the batch fermentation (in two phases, I and II) for 72 h and the data was plotted between total sugar concentration vs biogas produced. Biogas production was monitored with initial sugar (hexose) concentration being 32 g/L at pH 7.0 in phase I (from 0 h to 48 h) with 20% inoculum (18 h grown). At the start of phase II i.e. 48 h after start of the experiment, 100 mL of inoculum (12.5% v/v) (18 h grown) was introduced into the spent media (800 mL) and pH value was set at 6.5 in the same batch reactor. From the graph (Fig. 5) it can be inferred that at the time of inoculation the total sugar concentration was maximum with a value of 32 g/L and then it started declining gradually with increase in biogas production after adjusting the pH from 7.0 to pH 6.5 in the second phase until completion of the experiment (72 h). From the graph it can be observed that the biogas production increased with decrease in total sugar concentration.

### Biomass concentration

**Figure 6:**
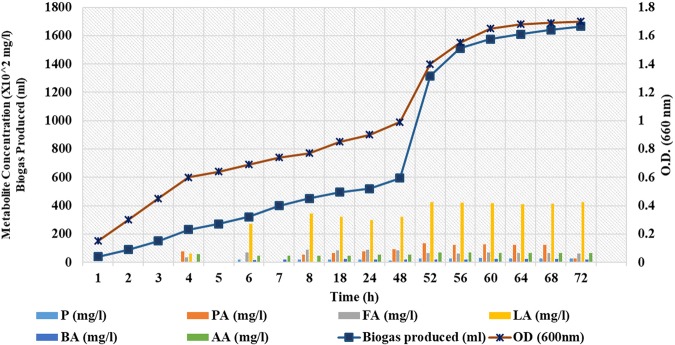
Kinetics of Biogas production (*p* < 0.05) and metabolites (*p* < 0.05) with cell growth during two phases (pH 7.0 and 6.5) in the batch fermentation at 37 °C from *L*. *acidophilus*.

The biomass concentration/cell growth was measured by taking absorbance at 600 nm after regular intervals of time and graph between time (h) and absorbance was plotted and is represented in Fig. (6). From the plot it can be inferred that the biomass concentration exhibited diauxic pattern that kept on increasing at a lower rate and reached maximum at 48 h and start growing further in Phase II after the reintroduction of inoculum at 48 h, the biomass concentration reached maximum at 64 h.

### Biogas production

The culture was observed for production of biogas from the time of initiation of the experiment and a graph between volume of biogas produced vs time was plotted as represented in Fig. 5. From the graph it can be observed that the biogas produced was minimum with a value of 40 mL just after the inoculation and then it increased during the bacterial growth and attained a value of 1665 mL at the end of the phase II indicating that the amount of biogas produced is proportional to the cell biomass concentration in the culture and varies in concentration dependent manner.

### Production of end metabolites

The end metabolites produced during bacterial growth which is directly related to the microbial metabolism influencing the fermentation pathways, were analysed for their concentrations using HPLC after sampling at different time intervals under the optimized conditions in batch experiment and metabolite concentrations were plotted against time as represented in Fig. (6). During sugar fermentation by *Lactobacillus*, the produced metabolites include lactate in homofermentation, lactate, acetate^33^ and propionate^34^ in heterofermentation. The other metabolites detected during the heterofermentation by *Lactobacillus* bacteria include pyruvate, formate and butyrate^35^ during the biogas production. The amounts of these metabolites varied in different studies owing to experiment conditions viz. inoculum age, inoculum amount, type of substrate used, pH, type of fermentation used^33–36^. In the present study the metabolites produced included pyruvic acid (PA), propionic acid (PrA), formic acid (FA), lactic acid (LA), butyric acid (BA) and acetic acid (AA) having maximum production at 60 h, 52 h, 24 h, 52 h, 64 h and 52 h respectively along with biogas production. During anaerobic fermentation pyruvate is further oxidized to acetyl-CoA by pyruvate: ferredoxin oxidoreductase (PFOR) complex as represented in the equation below.

$$Pyruvate+CoA+Fd\,\to Acetyl-CoA+FdH+C{O}_{2}$$

Further reduced ferredoxin is also generated in the reaction with NADH, with the reaction being catalysed by NADH: ferredoxin oxidoreductase (NFOR) as given below.

$$NADH+Fd\to NA{D}^{+}+FdH$$

In this metabolic pathway, hydrogen is released by hydrogenases responsible for catalysing proton reduction utilizing electrons from ferredoxin where the activity of both the involved enzymes is controlled by hydrogen concentration. The hydrogen partial pressure >60 Pa are reported to inhibit NFOR activity and result in production of non-gaseous end-products from acetyl-CoA including acetate, butyrate, ethanol, butanol and lactate. Similarly, PFOR is active at hydrogen concentrations lower than 3 × 10^4^ Pa. The theoretical maximum hydrogen yield during this type of fermentation is 4 ces of hydrogen per mole of hexose utilized. Whereas, during conversion of hexose into butyrate, the hydrogen yield drops to 2 moles per mole of hexose^34,35^. The results obtained in present study are in accordance with the previous reports of metabolite production during sugar fermentation using lactic acid bacteria.

The calculated values of the molar amounts of the end metabolites from the experiment were, AA (0.12 moles), PA (0.031 moles), BA (0.01 moles), LA (0.56 moles), FA (0.125 moles) and PrA (0.165 moles) from the 0.114 moles of hexose which are in accordance with the theoretical values. There is an increasing trend for formic acid synthesis as it is clear from graph (6) from 4–8 h corroborating the significant increase in biogas production through FHL pathway. However, which further increased with time from 8–12 hours. The flux of formic acid remains the same with reduction in lactic acid production where we achieved maximum biogas. However, production rate decreases after 8 hours of fermentation. Results are clearly showing that there is no significant rise after 10 hours of fermentation. However, accumulation of lactic acid increased (32.1%) from 10 h–48 h. From the graph it can be inferred that with time as lactic acid concentration increased, the production of biogas had also increased, which is in accordance with the results previously reported in other studies supporting that lactic acid produced in Phase I inhibited more production of lactic acid and supported more production of biogas in Phase II of fermentation^37^.

This finding demonstrated that *L*. *acidophilus* can be used for biohydrogen production as well as lactic acid, pyruvic acid, formic acid, acetic acid, butyric acid and propionic acid confirming the mixed acid fermentation under controlled anaerobic environment. Further the molar yield of biohydrogen produced was calculated with a value of 1 mol of H_2_ (67%) produced/mol of the hexose used. In a study, the maximum hydrogen yield obtained corresponds to 180 mL H_2_/gVS at 5% GLC (glycerol) with the maximum specific production rate value of 13 mL H_2_/(gVS.h)^38^. Different microorganisms yield H_2_ under specific conditions, such as use of light as energy source by microalgae to split water in production of H_2_ and cyanobacteria which utilize carbohydrates to store energy from photosynthesis and to produce H_2_ from water. In various studies different reactor configurations and reaction conditions have been analysed for bio-hydrogen production from food and other organic waste. In a study, waste paper has been utilized for biohydrogen gas production through dark fermentation. The highest amount of hydrogen gas obtained corresponds to 18.9 g/L of initial sugar concentration whereas sugar concentrations higher than 18.9 g/L resulted in inhibition of product formation^39^.

In another study anaerobic batch fermenter has been used in production of biohydrogen from cow solid waste under the specific hydrolysis conditions. The results exhibited a hydrogen production yield of 97 mL H_2_/g cow solid waste^40^. In a study where anaerobic fermentation of glucose by *Clostridium butyricum* yielded 2.02 mol H_2_/mol glucose have been reported^13^. Food waste hydrolysate has been used as a substrate with the production 1.97 mol H_2_/mol glucose in the batch system^2^. The comparison of biohydrogen produced utilizing various biowastes are shown in Table 1.

**Table 1:** Comparison of hydrogen production found in different studies by using wastes as the substrate.

S. no	Microbes	Wastes	Yield	References
1	*Thermoanaerobacterium*, *Caloribacterium*, and *Caldanaerobius Species* (thermophilic consortium)	Human waste stimulants, Wastewater, and Activated sludge	4 mmol H_2_/g (from human waste), 5.7 mmol H_2_/g (from waste water), and 2.2 mmol H_2_/g (from activated sludge)	^42^
2	*Rhodobacter capsulatus* JP91	Glucose	7.8 mol H_2_/mol glucose	^45^
3	Mixed culture	Sugar beet	198 mL H_2_ g^−1^ TOC	^47^
4	*Klebsiella peneumoniae* (Inoculum produced by isolating microorganism from aviary litter).	Brewery waste water	0.80–1.67 mol H_2_/mol glucose	^48^
5	*Thermotoga maritima* DSM 3109	Fruit and vegetable wastes	3.46 mol H_2_/mol	^49^
6	R. *capsulatus* DSM 1710, R. *capsulatus* YO3, R. *sphaeroides* O.U.001 and Rp. *palustris* DSM 127	Sugar beet molasses	12.7 ± 0.7 mol H_2_/mol sucrose 10.6 ± 0.4 mol H_2_/mol sucrose 9.4 ± 0.5 mol H_2_/mol sucrose and 19.0 ± 0.5 mol H_2_/mol sucrose respectively	^50^
7	Seed sludge	Dairy cow solid waste	500 mL H_2_/g total sugar	^40^
8	*Enterobacter aerogenes*	Rice straw	19.7 mL H_2_/g dry rice straw	^51^
9	Granule sludge	Vinasse	14.8 mL H_2_/g VS _substrate_	^52^
10	*Bacillus licheniformis* AP1	Kitchen waste	17.6 mmol H_2_/g COD	^53^
11	*Lactobacillus acidophilus*	Cheese waste	1 mol of H_2_ produced/mol of the hexose	This study

Hydrogen production from cheese processing wastewater by using mixed microbial cultures in anaerobic fermentation reached to a value of 10 mM/gCOD^41^ while using human wastes and activated sludge 2.186–3.999 mmol H_2_/g of waste has been obtained by thermophilic bacteria^42^. Production of 2.74 mol H_2_/mol lactose has been reported by *E*. *coli* using cheese whey as substrate^43^. In another study by using cheese whey and glucose as substrate, 1.33 mol hydrogen/mol lactose^44^ and 7.8 mol H_2_/mol glucose have been achieved respectively^45^. Using cheese whey powder (CWP) 1.03 mol H_2_/mol glucose has been obtained by thermophilic dark fermentation^46^. From the comparison studies, it can be inferred that biohydrogen produced in our designed experiment using non-pathogenic food grade bacteria with different parameters is within values reported by others.

## Conclusion

In comparison to the lipid, protein and cellulose components, the carbohydrate fraction in food waste plays a significant role in the hydrolysis step during anaerobic degradation. In present study the role of *Lactobacillus* was observed for biogas production in fed batch reactor using anaerobic fermentation. Among all the bacterial cultures under study, *L*. *acidophilus* was observed to have maximum biogas production value. For *L*. *acidophilus*, it was observed that under optimized inoculum conditions such as inoculum of 18 h age, and 12.5 mL of inoculum per 100 mL (12.5% v/v) of culture at pH value of 6.5, maximum biogas (1665 mL) was produced after 72 hours of incubation. Along with the biogas other valuable organic acids including pyruvate, propionate, acetate, lactate, formate and butyrate were also produced during anaerobic metabolism. Dark fermentation of food waste for biogas generation exhibits the potential to create an impact on the global energy market for the production of energy from a cheap and renewable carbon source. The isolation of viable and nonpathogenic LAB (lactic acid bacteria) cells for fermentative biogas production from cheese whey indicated that the LAB are capable of surviving in reactor conditions and can influence biogas production.
